# Ischemic stroke in a patient with EGFR‐mutated non–small‐cell lung cancer after treatment with ramucirumab

**DOI:** 10.1111/1759-7714.14701

**Published:** 2022-10-28

**Authors:** Guo‐Yu Chen, Wen‐Chien Cheng, Chih‐Yen Tu

**Affiliations:** ^1^ Department of Internal Medicine China Medical University Hospital Taichung Taiwan; ^2^ Division of Pulmonary and Critical Care Medicine, Department of Internal Medicine China Medical University Hospital Taichung Taiwan

**Keywords:** adverse effect, anti‐vascular endothelial growth factor receptor 2 monoclonal antibody, cerebral ischemia

## Abstract

Vascular endothelial growth factor (VEGF) inhibitors have been widely investigated in the last 10 years, with particular attention paid to their adverse effects because of their efficacy in improving cancer patient survival. Previous research primarily focused on the monoclonal anti‐vascular endothelial growth factor antibody bevacizumab and its adverse outcomes. Reports show a higher risk of ischemic stroke, one of the most concerning clinically relevant events, after treatment with bevacizumab. However, few studies have examined the relationship between anti‐VEGF receptor 2 monoclonal antibody ramucirumab and its adverse events. This article presents the case of a non–small‐cell lung cancer patient who experienced a new ischemic stroke after treatment with ramucirumab. The findings suggest that further studies may be necessary to investigate the relationship between ramucirumab and the risk of ischemic stroke.

## Graphical abstract

A case of a non–small‐cell lung cancer patient harboring epidermal growth factor receptor mutation with multiple brain metastasis experienced a new ischemic stroke after treatment with ramucirumab.

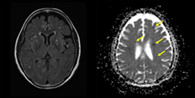

## INTRODUCTION

Research interest is growing in various adverse events of anti‐vascular endothelial growth factor (anti‐VEGF) medication because of its wide use and efficacy in improving the survival of cancer patients.^1^, ^2^, ^3^ Myocardial infarction, hypertension, and new‐onset proteinuria have been reported as adverse events.^4^ Ischemic stroke is also one of the most concerning clinically relevant adverse events.^4^, ^5^, ^6^, ^7^, ^8^, ^9^ However, the relationship between anti‐VEGF agents and the incidence of cerebral ischemia is still controversial.^4^, ^5^, ^6^, ^7^, ^8^, ^9^ Bevacizumab and ramucirumab are vascular endothelial growth factor (VEGF) signaling pathway inhibitors and are indicated in the treatment of non–small‐cell lung cancer (NSCLC) patients.^10^, ^11^ Although treatment with bevacizumab has been reported to increase the risk of cerebral ischemia,^9^ stroke cases have rarely been mentioned after the use of ramucirumab. To our knowledge, only one case has been reported of an 82‐year‐old male with metastatic gastric cancer suffering from multifocal ischemic stroke after treatment with four doses of ramucirumab.^12^ We present the first case of an ischemic stroke in an NSCLC patient after treatment with ramucirumab.

## CASE REPORT

**FIGURE 1:**
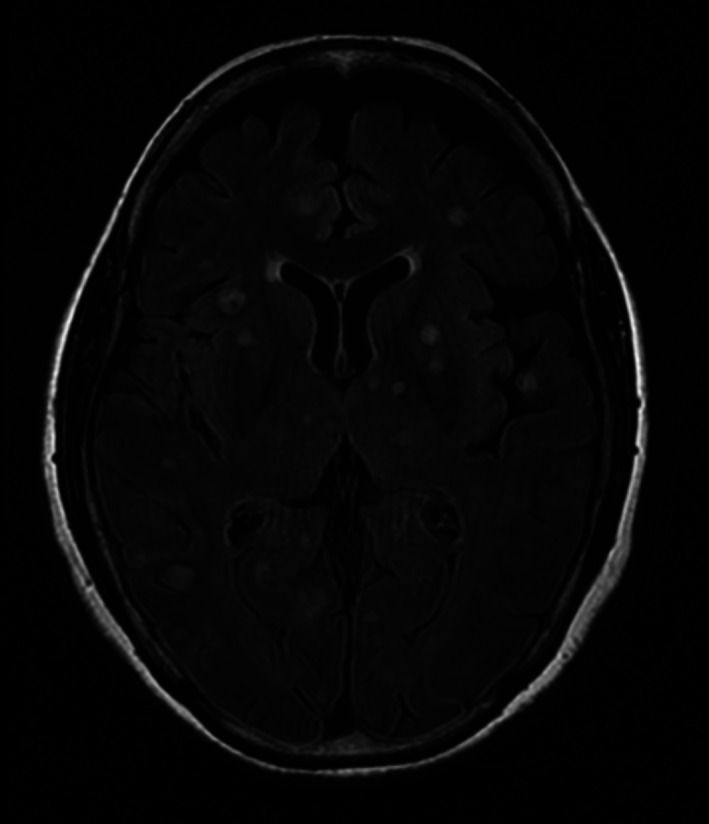
T_2_‐weighted fluid attenuated inversion recovery (FLAIR) brain magnetic resonance imaging for initial cancer status evaluation revealed multiple hyperintense lesions in the bilateral hemispheres and suspected non–small‐cell lung cancer with brain metastasis.

A 67‐year‐old female diagnosed with NSCLC harboring the EGFR‐L858R mutation, with liver, spine, right rib, bilateral adrenal gland, and brain metastases (Figure 1), visited our chest outpatient department because of frequent dizziness and falls. Her past medical history included type II diabetes mellitus with metformin control and asthma for 30 years. She initially received targeted therapy with second‐generation epidermal growth factor receptor (EGFR) tyrosine kinase inhibitors (TKI) plus ramucirumab for treatment. After receiving ramucirumab for approximately 1.5 months, she began suffering from persistent dizziness and frequent falls.

**FIGURE 2:**
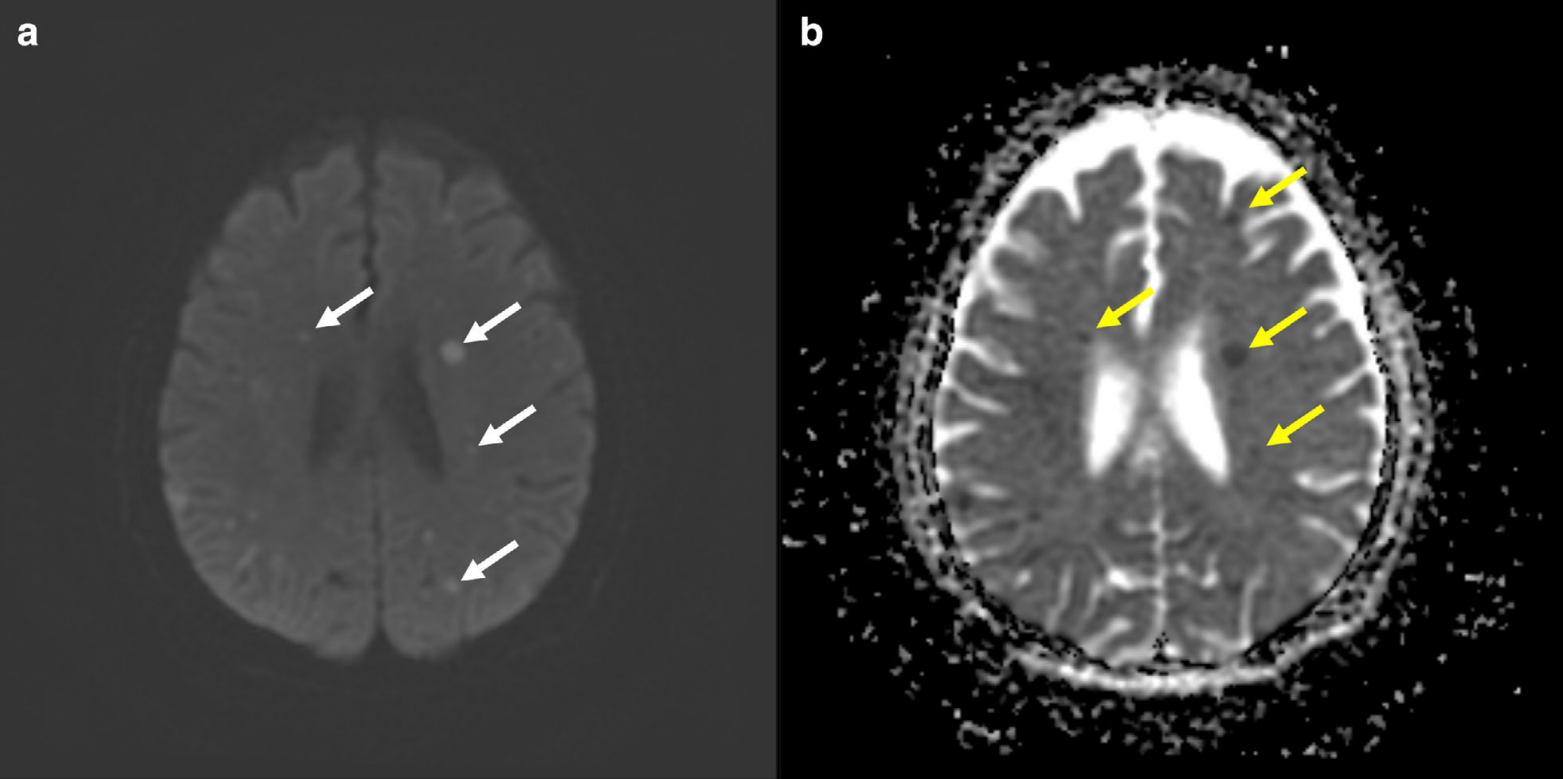
Sequential diffusion‐weighted imaging (a) and the apparent diffusion coefficient (b) of the brain magnetic resonance imaging showed an increased multifocal signal because of diffusion restriction and a corresponding decrease in signal intensity, respectively. The result is compatible with ischemic stroke‐related cytotoxic edema.

On examination, she appeared chronically ill, but was still alert. She could only say a single word. Her vital signs were stable. A physical examination showed medial side deviation when performing an extraocular muscle function test (the provider moved the object to the left side), poor gag reflex, decreased upper limb muscle power (2 points), mild flattening of the left nasolabial fold, and a positive Babinski sign on the left side. Laboratory tests revealed leukocytosis with neutrophil predominance (WBC, 17,600/μL; neutrophil, 86.8%). Repeated brain magnetic resonance imaging performed because of suspected progression of the disease disclosed partial remission of diffuse intracranial metastatic tumors with residual tumors in the midbrain, cerebellum, bilateral basal ganglia, and bilateral hemispheres of the cerebrum, but suspected diffuse embolic acute infarctions in the left thalamus, bilateral basal ganglia, bilateral corona radiata, and bilateral temporal, bilateral frontal, bilateral parietal, and bilateral occipital lobes (Figure 2). The electrocardiogram showed no arrhythmia. Transcranial Doppler sonography results were within normal limits except for very mild atherosclerotic changes in the bilateral bulb. Echocardiography revealed preserved left and right ventricular contractility with normal chamber size and no abnormality in left ventricular regional wall motion. The patient then received rehabilitation and acupuncture for post‐stroke care.

## DISCUSSION

To our knowledge, this is the first case report detailing a new‐onset ischemic stroke in an NSCLC patient after ramucirumab treatment. A previous case report described a patient with metastatic gastric cancer who suffered from an ischemic stroke after treatment with ramucirumab.^12^ However, these cases are rarely reported. Bevacizumab has been well investigated in previous studies that discussed the relationship between angiogenesis inhibitors and the risk of arterial thromboembolism (ATE). In a meta‐analysis that evaluated cardiovascular adverse effects in cancer patients after treatment with bevacizumab, Totzek et al.^9^ reported a higher risk of developing cerebral ischemia in the bevacizumab group (relative risk [RR], 3.11; 95% CI, 1.46–6.65; *p* = 0.003). Another meta‐analysis of 77 Phase III trials that primarily included bevacizumab also showed a higher odds ratio for ATE (1.52 [95% CI, 1.17–1.98]).^5^ Nevertheless, only a few studies have targeted ramucirumab for evaluation. A report that examined the incidence of adverse events, possibly because of VEGF signaling pathway inhibition by ramucirumab, revealed 38 (1.4%) and 40 (1.8%) cases of all‐grade ATE in the ramucirumab and control arms, respectively (RR, 0.8; 95% CI, 0.5–1.3).^6^ However, patients who had a cerebrovascular accident or transient ischemic attack within 6 to 12 months before randomization were excluded in five of the six trials included in the study, and therefore, whether or not patients were better selected to reduce the risk of ATE is unknown.^6^ In our case, the patient had long‐term diabetes mellitus, but the condition was well controlled by medication. Her cancer status was a partial response of tumor shrinkage noted by imaging. Although both are considered risk factors for stroke, the conditions were relatively stable; therefore, their effects might have been slight.

In conclusion, we report a new‐onset ischemic stroke in an NSCLC patient after treatment with ramucirumab. The previous study targeting ramucirumab may be limited because of patient inclusion criteria.^6^ Therefore, further extensive studies are necessary to investigate the relationship between ramucirumab and the risk of ischemic stroke.

## CONFLICT OF INTEREST

The authors declare that they have no competing interests.

## INFORMED CONSENT

Informed consent was obtained from the patient in this study.

## Data Availability

The data that support the findings of this study are available on request from the corresponding author, Wen‐Chien Cheng. The data are not publicly available because they include information that could compromise the privacy of the patient.

## References

[1] Garon EB , Ciuleanu TE , Arrieta O , Prabhash K , Syrigos KN , Goksel T , et al. Ramucirumab plus docetaxel versus placebo plus docetaxel for second‐line treatment of stage IV non‐small‐cell lung cancer after disease progression on platinum‐based therapy (REVEL): a multicentre, double‐blind, randomised phase 3 trial. Lancet. 2014;384(9944):665–73. 24933332 10.1016/S0140-6736(14)60845-X

[2] Nakagawa K , Garon EB , Seto T , Nishio M , Ponce Aix S , Paz‐Ares L , et al. Ramucirumab plus erlotinib in patients with untreated, EGFR‐mutated, advanced non‐small‐cell lung cancer (RELAY): a randomised, double‐blind, placebo‐controlled, phase 3 trial. Lancet Oncol. 2019;20(12):1655–69. 31591063 10.1016/S1470-2045(19)30634-5

[3] Sandler A , Gray R , Perry MC , Brahmer J , Schiller JH , Dowlati A , et al. Paclitaxel‐carboplatin alone or with bevacizumab for non‐small‐cell lung cancer. N Engl J Med. 2006;355(24):2542–50. 17167137 10.1056/NEJMoa061884

[4] Faruque LI , Lin M , Battistella M , Wiebe N , Reiman T , Hemmelgarn B , et al. Systematic review of the risk of adverse outcomes associated with vascular endothelial growth factor inhibitors for the treatment of cancer. PLoS One. 2014;9(7):e101145. 24988441 10.1371/journal.pone.0101145 PMC4079504

[5] Abdel‐Qadir H , Ethier JL , Lee DS , Thavendiranathan P , Amir E . Cardiovascular toxicity of angiogenesis inhibitors in treatment of malignancy: a systematic review and meta‐analysis. Cancer Treat Rev. 2017;53:120–7. 28104567 10.1016/j.ctrv.2016.12.002

[6] Arnold D , Fuchs CS , Tabernero J , Ohtsu A , Zhu AX , Garon EB , et al. Meta‐analysis of individual patient safety data from six randomized, placebo‐controlled trials with the antiangiogenic VEGFR2‐binding monoclonal antibody ramucirumab. Ann Oncol. 2017;28(12):2932–42. 28950290 10.1093/annonc/mdx514 PMC5834052

[7] Gu B , Gao WC , Chu HJ , Gao J , Fu Z , Ding H , et al. Adverse events risk associated with anti‐VEGFR agents in the treatment of advanced nonsmall‐cell lung cancer: a meta‐analysis. Medicine. 2016;95(48):e3752. 27902583 10.1097/MD.0000000000003752 PMC5134808

[8] Guo C , Yan C , Qu L , du R , Lin J . The efficacy and toxicity of angiogenesis inhibitors for ovarian cancer: a meta‐analysis of randomized controlled trials. Arch Gynecol Obstet. 2021;303(2):285–311. 33222040 10.1007/s00404-020-05865-z

[9] Totzeck M , Mincu RI , Rassaf T . Cardiovascular adverse events in patients with cancer treated with bevacizumab: a meta‐analysis of more than 20 000 patients. J Am Heart Assoc. 2017;6(8):e006278. 28862931 10.1161/JAHA.117.006278 PMC5586462

[10] Bevacizumab [package insert on the internet]. South San Francisco, CA: Genentech, Inc.; 2004 [updated 2021 Jan; cited 2022 Aug 29]. Available from: https://www.gene.com/download/pdf/avastin_prescribing.pdf

[11] Cyramza [package insert on the internet]. Indianapolis, IN: Eli Lilly and Company.; 2014 [updated 2020 May, cited 2022 Aug 29]. Available from: https://www.accessdata.fda.gov/drugsatfda_docs/label/2020/125477s034lbl.pdf

[12] Christiansen ME , Ingall T , Lew EC , Ramanathan RK , Paripati HR . A case report of ischemic stroke in a patient with metastatic gastric cancer secondary to treatment with the vascular endothelial growth factor Receptor‐2 inhibitor ramucirumab. Case Rep Oncol. 2016;9(2):317–20. 27462231 10.1159/000446695 PMC4939672

